# Supplementary figures and images for: The use of newborn foot length to identify low birth weight and preterm babies in Papua New Guinea: A diagnostic accuracy study
Source: PLOS Glob Public Health. 2023 Jun 21;3(6):e0001924. doi: 10.1371/journal.pgph.0001924 (PMC10284404; doi:10.1371/journal.pgph.0001924)

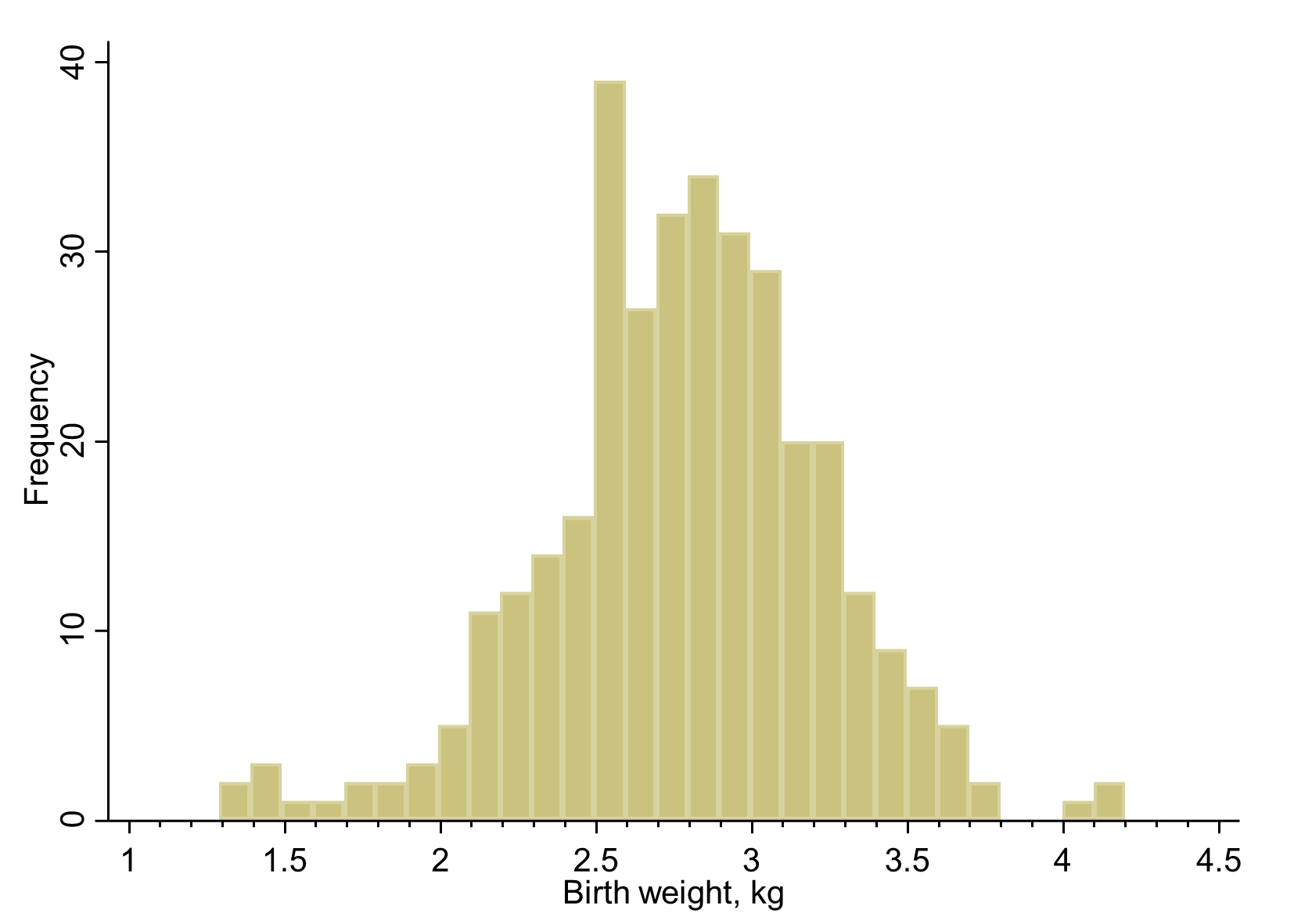

Supplement: S1 Fig — (TIF) [file pgph.0001924.s002.tif]

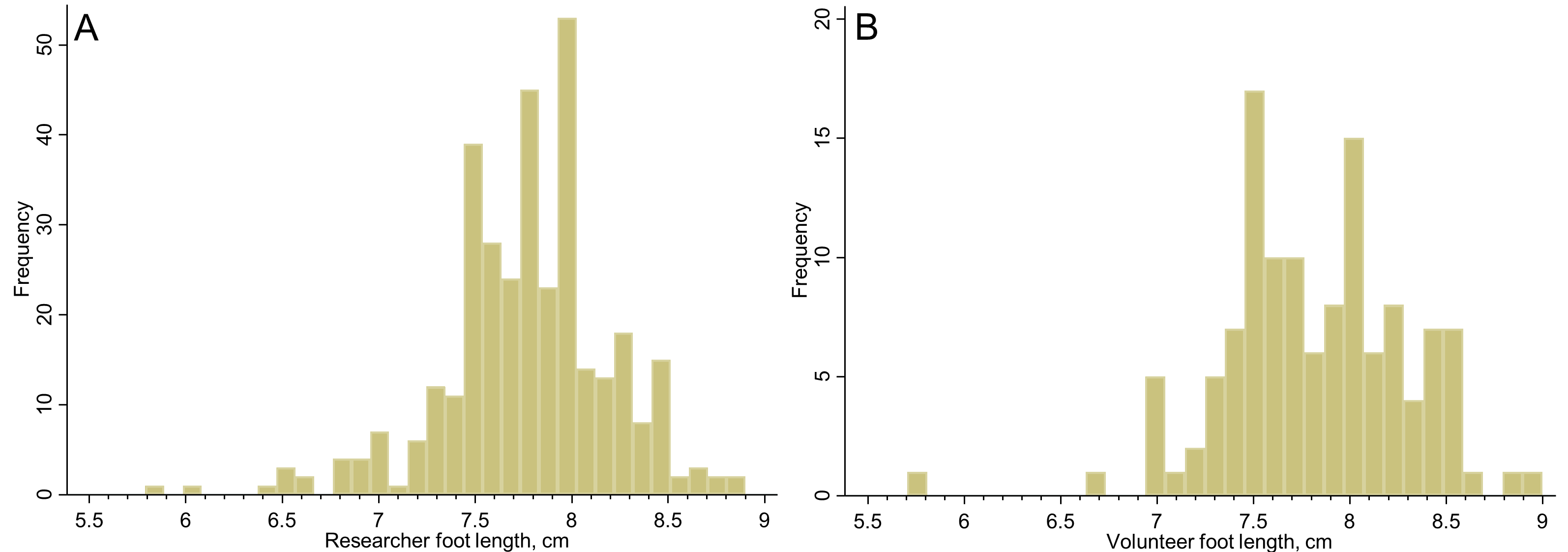

Supplement: S2 Fig — Panel A, researcher (N = 342), B, volunteer (N = 123). (TIF) [file pgph.0001924.s003.tif]
