# Supplementary material for: The use of newborn foot length to identify low birth weight and preterm babies in Papua New Guinea: A diagnostic accuracy study
Source: PLOS Glob Public Health. 2023 Jun 21;3(6):e0001924. doi: 10.1371/journal.pgph.0001924 (PMC10284404; doi:10.1371/journal.pgph.0001924)
Supplement: S1 Table — Abbreviations: CI, confidence interval; LHR, likelihood ratio; PV, predictive value. (DOCX) [file pgph.0001924.s004.docx]

**S1 Table.** Receiver operating curve statistics for accuracy of newborn foot length to classify low birth weight, 1 mm increments (N=342)

| **Low birth weight (<2.50 kg vs ≥2.50 kg)** | **True positive** | **False negative** | **False positive** | **True negative** | **Sensitivity, % (95% CI)** | **Specificity, % (95% CI)** | **Positive PV, % (95% CI)** | **Negative PV, % (95% CI)** | **Positive LHR, % (95% CI)** | **Negative LHR, % (95% CI)** | **AUC, % (95% CI)** | **Total, N** |
| --- | --- | --- | --- | --- | --- | --- | --- | --- | --- | --- | --- | --- |
| Researcher, overall |  |  |  |  |  |  |  |  |  |  | 87.0 (82.8-90.2) |  |
| Foot length (<5.8 cm) | 0 | 72 | 0 | 270 | 0.0 (0.0-5.1) | 100.0 (98.6-100.0) | .. | 78.9 (74.3-82.9) | .. | 1.0 (1.0-1.0) | 50.0 (44.6-55.4) | 342 |
| Foot length (<5.9 cm) | 1 | 71 | 0 | 270 | 1.4 (0.2-7.5) | 100.0 (98.6-100.0) | 100.0 (20.7-100.0) | 79.2 (74.6-83.2) | .. | 1.0 (1.0-1.0) | 50.7 (45.2-56.0) | 342 |
| Foot length (<6 cm) | 1 | 71 | 0 | 270 | 1.4 (0.2-7.5) | 100.0 (98.6-100.0) | 100.0 (20.7-100.0) | 79.2 (74.6-83.2) | .. | 1.0 (1.0-1.0) | 50.7 (45.2-56.0) | 342 |
| Foot length (<6.1 cm) | 2 | 70 | 0 | 270 | 2.8 (0.8-9.6) | 100.0 (98.6-100.0) | 100.0 (34.2-100.0) | 79.4 (74.8-83.4) | .. | 1.0 (0.9-1.0) | 51.4 (46.0-56.9) | 342 |
| Foot length (<6.2 cm) | 2 | 70 | 0 | 270 | 2.8 (0.8-9.6) | 100.0 (98.6-100.0) | 100.0 (34.2-100.0) | 79.4 (74.8-83.4) | .. | 1.0 (0.9-1.0) | 51.4 (46.0-56.9) | 342 |
| Foot length (<6.3 cm) | 2 | 70 | 0 | 270 | 2.8 (0.8-9.6) | 100.0 (98.6-100.0) | 100.0 (34.2-100.0) | 79.4 (74.8-83.4) | .. | 1.0 (0.9-1.0) | 51.4 (46.0-56.9) | 342 |
| Foot length (<6.4 cm) | 2 | 70 | 0 | 270 | 2.8 (0.8-9.6) | 100.0 (98.6-100.0) | 100.0 (34.2-100.0) | 79.4 (74.8-83.4) | .. | 1.0 (0.9-1.0) | 51.4 (46.0-56.9) | 342 |
| Foot length (<6.5 cm) | 3 | 69 | 0 | 270 | 4.2 (1.4-11.5) | 100.0 (98.6-100.0) | 100.0 (43.8-100.0) | 79.6 (75.0-83.6) | .. | 1.0 (0.9-1.0) | 52.1 (46.6-57.4) | 342 |
| Foot length (<6.6 cm) | 7 | 65 | 0 | 270 | 9.7 (4.8-18.7) | 100.0 (98.6-100.0) | 100.0 (64.6-100.0) | 80.6 (76.0-84.5) | .. | 0.9 (0.8-1.0) | 54.9 (49.5-60.3) | 342 |
| Foot length (<6.7 cm) | 7 | 65 | 0 | 270 | 9.7 (4.8-18.7) | 100.0 (98.6-100.0) | 100.0 (64.6-100.0) | 80.6 (76.0-84.5) | .. | 0.9 (0.8-1.0) | 54.9 (49.5-60.3) | 342 |
| Foot length (<6.8 cm) | 7 | 65 | 0 | 270 | 9.7 (4.8-18.7) | 100.0 (98.6-100.0) | 100.0 (64.6-100.0) | 80.6 (76.0-84.5) | .. | 0.9 (0.8-1.0) | 54.9 (49.5-60.3) | 342 |
| Foot length (<6.9 cm) | 11 | 61 | 0 | 270 | 15.3 (8.8-25.3) | 100.0 (98.6-100.0) | 100.0 (74.1-100.0) | 81.6 (77.0-85.4) | .. | 0.8 (0.8-0.9) | 57.6 (52.2-62.9) | 342 |
| Foot length (<7 cm) | 15 | 57 | 0 | 270 | 20.8 (13.1-31.6) | 100.0 (98.6-100.0) | 100.0 (79.6-100.0) | 82.6 (78.1-86.3) | .. | 0.8 (0.7-0.9) | 60.4 (55.1-65.7) | 342 |
| Foot length (<7.1 cm) | 22 | 50 | 2 | 268 | 30.6 (21.1-42.0) | 99.3 (97.3-99.8) | 91.7 (74.2-97.7) | 84.3 (79.9-87.9) | 41.2 (9.9-171.3) | 0.7 (0.6-0.8) | 64.9 (59.6-70.0) | 342 |
| Foot length (<7.2 cm) | 27 | 45 | 2 | 268 | 37.5 (27.2-49.0) | 99.3 (97.3-99.8) | 93.1 (78.0-98.1) | 85.6 (81.3-89.1) | 50.6 (12.3-207.9) | 0.6 (0.5-0.8) | 68.4 (63.2-73.3) | 342 |
| Foot length (<7.3 cm) | 28 | 44 | 3 | 267 | 38.9 (28.5-50.4) | 98.9 (96.8-99.6) | 90.3 (75.1-96.7) | 85.9 (81.5-89.3) | 35.0 (11.0-111.9) | 0.6 (0.5-0.7) | 68.9 (63.8-73.9) | 342 |
| Foot length (<7.4 cm) | 37 | 35 | 5 | 265 | 51.4 (40.1-62.6) | 98.1 (95.7-99.2) | 88.1 (75.0-94.8) | 88.3 (84.2-91.5) | 27.8 (11.3-68.0) | 0.5 (0.4-0.6) | 74.8 (69.9-79.4) | 342 |
| Foot length (<7.5 cm) | 40 | 32 | 15 | 255 | 55.6 (44.1-66.5) | 94.4 (91.0-96.6) | 72.7 (59.8-82.7) | 88.9 (84.7-92.0) | 10.0 (5.9-17.0) | 0.5 (0.4-0.6) | 75.0 (70.2-79.6) | 342 |
| Foot length (<7.6 cm) | 54 | 18 | 64 | 206 | 75.0 (63.9-83.6) | 76.3 (70.9-81.0) | 45.8 (37.0-54.7) | 92.0 (87.7-94.9) | 3.2 (2.5-4.1) | 0.3 (0.2-0.5) | 75.6 (70.8-80.2) | 342 |
| Foot length (<7.7 cm) | 61 | 11 | 82 | 188 | 84.7 (74.7-91.2) | 69.6 (63.9-74.8) | 42.7 (34.8-50.9) | 94.5 (90.4-96.9) | 2.8 (2.3-3.4) | 0.2 (0.1-0.4) | 77.2 (72.4-81.5) | 342 |
| Foot length (<7.8 cm) | 61 | 11 | 83 | 187 | 84.7 (74.7-91.2) | 69.3 (63.5-74.5) | 42.4 (34.6-50.5) | 94.4 (90.3-96.9) | 2.8 (2.2-3.4) | 0.2 (0.1-0.4) | 77.0 (72.1-81.3) | 342 |
| Foot length (<7.9 cm) | 66 | 6 | 123 | 147 | 91.7 (83.0-96.1) | 54.4 (48.5-60.3) | 34.9 (28.5-42.0) | 96.1 (91.7-98.2) | 2.0 (1.7-2.3) | 0.2 (0.1-0.3) | 73.1 (68.1-77.7) | 342 |
| Foot length (<8 cm) | 69 | 3 | 144 | 126 | 95.8 (88.5-98.6) | 46.7 (40.8-52.6) | 32.4 (26.5-38.9) | 97.7 (93.4-99.2) | 1.8 (1.6-2.0) | 0.1 (0.0-0.3) | 71.3 (66.2-76.1) | 342 |
| Foot length (<8.1 cm) | 71 | 1 | 193 | 77 | 98.6 (92.5-99.8) | 28.5 (23.5-34.2) | 26.9 (21.9-32.5) | 98.7 (93.1-99.8) | 1.4 (1.3-1.5) | 0.0 (0.0-0.3) | 63.6 (58.1-68.6) | 342 |
| Foot length (<8.2 cm) | 71 | 1 | 221 | 49 | 98.6 (92.5-99.8) | 18.1 (14.0-23.2) | 24.3 (19.7-29.5) | 98.0 (89.5-99.6) | 1.2 (1.1-1.3) | 0.1 (0.0-0.5) | 58.4 (53.1-63.8) | 342 |
| Foot length (<8.3 cm) | 71 | 1 | 221 | 49 | 98.6 (92.5-99.8) | 18.1 (14.0-23.2) | 24.3 (19.7-29.5) | 98.0 (89.5-99.6) | 1.2 (1.1-1.3) | 0.1 (0.0-0.5) | 58.4 (53.1-63.8) | 342 |
| Foot length (<8.4 cm) | 72 | 0 | 246 | 24 | 100.0 (94.9-100.0) | 8.9 (6.0-12.9) | 22.6 (18.4-27.6) | 100.0 (86.2-100.0) | 1.1 (1.1-1.1) | 0.0 (.-.) | 54.4 (48.9-59.8) | 342 |
| Foot length (<8.5 cm) | 72 | 0 | 246 | 24 | 100.0 (94.9-100.0) | 8.9 (6.0-12.9) | 22.6 (18.4-27.6) | 100.0 (86.2-100.0) | 1.1 (1.1-1.1) | 0.0 (.-.) | 54.4 (48.9-59.8) | 342 |
| Foot length (<8.6 cm) | 72 | 0 | 261 | 9 | 100.0 (94.9-100.0) | 3.3 (1.8-6.2) | 21.6 (17.5-26.4) | 100.0 (70.1-100.0) | 1.0 (1.0-1.1) | 0.0 (.-.) | 51.7 (46.3-57.2) | 342 |
| Foot length (<8.7 cm) | 72 | 0 | 265 | 5 | 100.0 (94.9-100.0) | 1.9 (0.8-4.3) | 21.4 (17.3-26.1) | 100.0 (56.6-100.0) | 1.0 (1.0-1.0) | 0.0 (.-.) | 50.9 (45.4-56.3) | 342 |
| Foot length (<8.8 cm) | 72 | 0 | 266 | 4 | 100.0 (94.9-100.0) | 1.5 (0.6-3.7) | 21.3 (17.3-26.0) | 100.0 (51.0-100.0) | 1.0 (1.0-1.0) | 0.0 (.-.) | 50.7 (45.4-56.3) | 342 |
| Foot length (<8.9 cm) | 72 | 0 | 270 | 0 | 100.0 (94.9-100.0) | 0.0 (0.0-1.4) | 21.1 (17.1-25.7) |  | 1.0 (1.0-1.0) | .. | 50.0 (44.6-55.4) | 342 |

Abbreviations: CI, confidence interval; LHR, likelihood ratio; PV, predictive value
