# Supplementary material for: The use of newborn foot length to identify low birth weight and preterm babies in Papua New Guinea: A diagnostic accuracy study
Source: PLOS Glob Public Health. 2023 Jun 21;3(6):e0001924. doi: 10.1371/journal.pgph.0001924 (PMC10284404; doi:10.1371/journal.pgph.0001924)
Supplement: S1 Protocol — (DOCX) [file pgph.0001924.s006.docx]

The use of newborn foot length to identify preterm birth and low birth weight babies in rural Papua New Guinea

**MMSc Candidate**

Dr Alice Mengi, Research Paediatric Registrar, PNG Institute of Medical Research, PNG

**Supervisors**

Prof Nicola Low, Professor of Epidemiology and Public Health, University of Bern, Switzerland

Dr Moses Laman, Head of PNGIMR Vector Borne Diseases Unit, PNG and Adjunct Professor, School of Medicine and Health Sciences, University of PNG

Dr Michaela Riddell, Senior WANTAIM Trial Coordinator, PNG

Dr Lisa Vallely, Principal Investigator, WANTAIM Trial, PNG

Dr Jimmy Aipit, Senior Consultant and Head of Paediatric unit Modilon General Hospital, PNG

# Background

Every year over 4 million babies die in the first four weeks of life (the neonatal period) globally. Of these deaths, three million occur in the first seven days (early neonatal period).1-3 Ninety-eight percent of all neonatal deaths occur in developing countries where the risk of neonatal death is six times higher than in developed countries.1 Neonatal death rates are high in remote rural areas, where access to maternal and newborn health services, including skilled health professionals, is limited.4 The most common causes of early neonatal deaths are complications of pregnancy or childbirth, preterm birth, congenital malformations and infections or neonatal sepsis.1 Small for age or low birth weight infants are at higher risk of dying from infections than normal birth weight or appropriate for age infants.5 Preterm birth (babies born at less than 37 completed weeks gestation) and low birth weight (infants born weighing less than 2500g; <1500g birthweight is defined as very low birth weight) are two main factors that significantly contribute to neonatal mortality.1 Preterm birth is associated with delayed development, but evidence about the outcomes of those surviving the neonatal period is scarce.6 A systematic review that found 197 studies measuring outcomes of preterm birth in low and middle income countries found that only 16% were performed in community settings, 19% assessed developmental outcomes, none was done in the Pacific Region, and only 11% fulfilled criteria for high quality.6

Simple, affordable tools to assess gestational age and birth weight would help to identify newborns that may need referral for further assessment or urgent interventions. There are several methods for assessing gestational age during pregnancy, and this includes last menstrual period, fundal height, or ultrasound scan (USS) before 22 weeks of gestation. Neonatal gestational age can be estimated from either the Dubowitz (22 clinical factors)7 or Ballard score (12 clinical factors).8 However, a major challenge, particularly in resource-limited settings, is that for such diagnostic tools, skilled health workers, or complex equipment, or both, are usually needed to assess gestational age accurately.8

For women who give birth in rural and remote areas or in the absence of skilled healthcare personnel, preterm birth and low birth weight are difficult to assess and weighing scales are not always available. Several studies have examined a range of anthropometric measures, which can be assessed easily in resource-limited settings, as proxies for preterm birth and low birth weight.9-11 These studies compared the accuracy of these anthropometric measures with more accurate, reference standards (e.g. USS for gestational age and digital scales for birth weight). Some studies suggested that chest circumference, followed by either mid upper arm circumference (MUAC) or head circumference are highly predictive of low birth weight and preterm birth.9-12

Foot length is another measurement that correlates well in determining preterm birth and low birth weight in several countries in sub Saharan Africa and South Asia, but there have been no similar studies in the Pacific Region.13-20 Foot length is simple to measure with a ruler or tape measure, items which could be readily available in the community and health facility. Studies conducted to date have suggested different cut-offs for foot length that predict preterm birth or low birth weight, ranging from 6.8cm by Hadush et al. in Ethiopia (2017) to 8.0cm by Marchant et al. in Tanzania (2010 and 2014).12,18-19 However, these studies differed in the way in which gestational age was assessed, as well as in the definitions of preterm birth and low birth weight and the method for measuring foot length.8-16,18-21 Nabiwemba et al. found that a hard plastic ruler was more accurate than a flexible tape measure or a printed foot print.15

Papua New Guinea (PNG) had the highest infant mortality rate 50-59 per 1000 births in a 2014 Pacific Islands report.22 An estimated 56% of women in PNG give birth unsupervised, the majority in their villages.23 Health extension officers, nurses, nursing officers and community health workers are trained to measure fundal height during pregnancy to assess gestational age, and to take other anthropometric measurements. If simpler methods, such as foot length, could be used to assess gestational age and birth weight, village birth attendants or village reporters could be trained to do such measurements, which may facilitate referral of vulnerable newborns for further management and help to reduce neonatal and infant mortality in rural PNG.

The Women And Neonatal Trial of Antenatal Interventions and Management (WANTAIM) is a randomized cluster crossover trial of the effectiveness of point-of- care testing for sexually transmitted infections in pregnancy to improve birth outcomes. The prevention of adverse outcomes, such as preterm birth, low birth weight, neonatal eye infections and neonatal pneumonia should contribute to improved infant health. The trial enrols women attending antenatal clinics and includes assessment of gestational age by USS and neonatal examination within 72 hours of birth. This setting allows for assessment of both anthropometric measurements, including foot length, within 72 hours of birth and follow up of physical growth and development outcomes beyond the neonatal period in a community-enrolled sample. Such information will contribute to evidence needed to assess the burden of disease due to preterm birth and low birth weight.6

# Study Objectives

The primary objective of this Neonatal Foot Length (Neofoot) study is to determine the diagnostic accuracy of foot length measurement to predict preterm birth and low birth weight in rural PNG.

Secondary objectives are to:

1. Compare foot length, MUAC and head circumference as predictors of preterm birth and low birth weight
2. Validate a specific foot length cut-off or derive a new cut-off appropriate for PNG
3. Assess the feasibility of using foot length as a measure for preterm birth and low birth weight in the community and health centre by community reporters/volunteers and health centre staff.
4. Determine associations between foot length at birth, physical growth and development of the infant at 6 months, according to study arm.

# Material and Methods

## Study Design

This study will be a prospective cohort study.

## Study setting

The Neonatal Foot Length (Neofoot) study will be conducted as a sub-study of the Women and Newborn Trial of Antenatal Interventions and Management (WANTAIM) in three WANTAIM trial sites in Madang Province.

## Ethics

PNG Institute of Medical Research (PNGIMR) Institutional Review Board with have given ethics approval to this study with IRB number 1811(Appendix1). Ethics approval will also be obtained from Medical Research Advisory Council (MRAC) of the National Department of Health (NDoH) and the University of PNG (UPNG).

## Eligibility criteria

Women enrolled in the two WANTAIM sites will be eligible if they have complete data on USS assessment of gestational age and have a post-natal visit completed within 72 hours of birth. Women whose baby died in the perinatal period, or who have very sick babies or babies with congenital abnormalities that restrict team from collecting outcomes within 72 hours of birth, or who will be moving out of the catchment area within the next 6 months, will be excluded. Locator information for eligible participants, collected at WANTAIM enrolment will be updated.

## Procedures

### Staff training

Community reporters/volunteers and health centre staff will be trained by the paediatric research registrar to measure infant foot length accurately.

All other study-specific training, including measurement of MUAC, weight, length, head circumference, respiratory rate, and heart rate, and completion of Neofoot study CRFs will be conducted by the paediatric research registrar.

### Informed consent procedure

The informed consent procedure for the Neofoot study will be conducted by the paediatric research registrar after completion of the WANTAIM 72-hour post-natal assessment. After assessing literacy, the research registrar will explain the procedures of the Neofoot study to the parent/caregiver (Appendix 2a, English, Appendix 3a, Pidgin) and obtain written consent (Appendix 2b, English, Appendix 3b, Pidgin). If the parent/caregiver is assessed as illiterate, an independent literate witness will be present during the informed consent process to attest that the information was explained accurately, was apparently understood, and that consent was given voluntarily. The parent/caregiver will either put a written mark on the consent form or put their thumbprint onto the consent form. The witness will then sign and date the consent form.

### Post-natal 72 hours and 6 month assessments

At the first post-natal (72 hours) assessment, anthropometric measurements in addition to those collected for WANTAIM will be carried out, either at the health facility or at home. At this time, a follow-up visit will be planned for 6 months later, to take place at a health centre. Table 1 shows the measurements that will be collected at each assessment.

Foot length will be measured using a stiff clear plastic ruler. The right foot will be measured from the heel to the tip of the big toe.18

As part of the 72-hour post-natal assessment, the mother or care giver will also be given post-natal health information about immediate care of her newborn infant. The handheld baby book will be used as a guide to discuss essential care of the baby (cord care, keeping the baby warm, regular breast feeding, immunisation and well clinic schedules at 1 month, 2 months, 3 months, 4 months, 5 months and 6 months), recognising danger signs in newborn and infants and actions to take in the event of these.

The 6-month follow-up visit will be conducted at a health centre or at home if the mother/care giver does not attend the clinic visit. The research registrar will be responsible for assessing the baby and collecting the anthropometric and development data (Table 1) and recording it in the study-specific CRF. Information about growth assessments using World Health Organization growth charts, immunisations, illnesses and hospital admissions in the period between the two visits will be obtained from discussion with the mother/care giver and from the baby book and recorded in the study specific CRF. If the registrar is concerned about a particular aspect of the infant’s development, or if the infant is unwell (including undernourished), the paediatric registrar will follow routine procedures for referring that infant for paediatric review either as an outpatient or an emergency, as the situation warrants. In addition, if the visit takes place in the home, the mother will be advised to attend the child health clinic to update immunisations, as required.

Table 1: Description of procedures at Neofoot study assessments within 72 hours and at 6 months

| **Procedures** | **Description or method** | **Person responsible** | **Neofoot enrolment** |
| --- | --- | --- | --- |
| Eligibility | Assess whether participant satisfies criteria to join the study | Researcher | X |
| Literacy check | Check if the participant is literate. So that steps such as an independent witness can help to ensure the parent fully understands the risk/benefits and requirements of participating tin the study | Researcher | X |
| Informed consent procedure (including flip chart) | Parent/carer of the newborn will be informed about the Neofoot study and procedure. If they wish to join, they will sign the consent form | Researcher | X |
|  | Weight is measured using digital scales | WANTAIM clinical staff |  |
| Foot length measurement | The right foot was measured from the heel to the end of the big toe, using a stiff clear plastic ruler. | Researcher | X |
|  |  |  |  |
|  |  | Volunteer | X |
|  |  |  |  |

Abbreviations: Women And Newborns Trial of Antenatal Intervention and Management

## Possible sources of bias in outcome measurements

Foot length measurement could be biased if the gestational age is known by the person undertaking the foot length measurement. This bias can be minimized by making the gestational age unavailable when foot length is measured. We will also measure foot length before going through other documents (client held record book, admission notes).

## Data management

All the data from this project will be managed by the Paediatric Research Registrar using paper based case record forms (CRF) to record the information at first postnatal (72hr) visit and 6 month postnatal visit. All CRFs will be designed in TELEform™ Elite version 10.5 (https://teleform.software.informer.com/10.5/). Completed CRFs will be checked on the day of completion and any errors, discrepancies or out of range values will be corrected and initialed by the Paediatric Research Registrar. Original copies of the CRF will be held in the PNGIMR WANTAIM Office in a locked filing cabinet accessible only by the Paediatric Research Registrar and the Trial Coordinator. Data entry and review will be ongoing throughout the entire trial period and will be conducted in accordance with WANTAIM study-specific SOPs. Completed CRFs will be electronically scanned at the PNGIMR WANTAIM Office, as completed using a Kodak ScanMate i940 portable scanner, into a computer using a Tagged Image File Format (TIFF). Data will be stored in a dedicated electronic folder prior to verification using TELEform™. A copy of the locally-held data will then be uploaded into a Microsoft SQL spreadsheet before importing into STATA (Stata 14.2, College Station, Texas, United States) for analysis. Electronic versions of the locked datasets and TIFF files for this study will be maintained as part of the PNGIMR’s research studies database. Access to the trial database will be limited to the trial Paediatric Research Registrar, and Principal Investigators. After completion of data analyses, copies of the final database and analytical datasets (neither of which will contain any subject identifying information) will be maintained on secure high-end computers located at the PNGIMR.

## Sample size

The maximum sample size will be determined by the number of women in WANTAIM who deliver during the study period. The required sample size is determined by the required precision around the estimates of sensitivity, the primary outcome. Based on projections of trial enrolment at the study clinics, an estimated 400 women will be invited to participate. If 95% have a 72-hour post-natal visit and 90% of mothers agree to foot length measurement and follow up, there will be 342 neonates. If sensitivity is 70-90%, 95% confidence intervals will be +/- 4 to 5%, which is an acceptable level of precision. If 80% of these infants are followed up at 6 months, we will have repeated anthropometric and physical development measurements on 274 infants. This sample size also ample statistical power for secondary outcomes, such as differences in the mean foot length at birth between infants who have or have not reached one or more developmental milestones. Assessment of the reliability of foot length measurements by community reporters will be measured using the kappa statistic (κ). Based on a published study,18 if agreement is fair between two raters’ measurements of a foot length <8cm, 143 pairs of observations are needed for an estimate of κ=0.4 and its 95% confidence interval. We will therefore enrol 150 consecutive babies for this assessment.

## Statistical analysis

I will examine the distributions of each variable. I will describe continuous variables using histograms, mean and standard deviation, and median and interquartile range. I will describe frequencies of categorical variables as percentages. I will conduct a descriptive analysis to examine associations with preterm delivery and low birth weight, including social and demographic factors (collected during WANTAIM assessments) and achievement of developmental milestones at 6 months and between study arms and achievement of developmental milestones at 6 months. I will assess the correlation between the gestational age at birth (measured by USS) and birthweight (measured using digital scales) and anthropometric measures (foot length, head circumference, MUAC) using Spearman’s rank correlation coefficient.

I will calculate diagnostic performance of foot length as: a) sensitivity (proportion of newborns correctly identified as preterm, low birthweight or very low birth weight); and b) specificity (proportion of newborns correctly identified as not preterm or not low birth weight) for 1mm increments in foot length. I will also calculate sensitivity and specificity for head circumference (1.0cm increments) and MUAC (1mm increments).8

To assess the diagnostic performance of each anthropometric measure as a predictor of preterm birth, low birth weight and very low birth weight, I will construct receiver operating characteristic (ROC) curves.24 The ROC curve is a graphical plot of sensitivity (vertical axis) against 1 minus specificity (horizontal axis) for each increment in measurement of the anthropometric measure. The best cut-off for each measure is the value at which the sensitivity and specificity are highest. I will then calculate the area under the curve (AUC, with 95% confidence intervals) as a proportion of the total area. The closer the AUC value is to one, the better the predictive value.

I will provide the best cut-off for the prediction of preterm birth, low birth weight and very low birth weight using foot length, head circumference and MUAC. I will recommend the most appropriate and simple measure based on my findings.

I will use linear regression to examine associations between the foot length at birth and achievement of developmental milestones, with multivariable models to control for potential confounding.

For the assessment of reliability of foot length measurement, I will calculate κ based on the expected and observed frequencies for the agreement of measurements and plot the difference between the raters against the average measurement of the two raters (Bland-Altman plot).24

# Ethical considerations

There are no major study-related risks for participants involved in this study. We will be using a stiff clear plastic ruler to measure the foot length and tape measure to measure head circumference and MUAC. This is a non-invasive assessment with no associated risk. At 6 months, babies will be assessed for their physical growth using scales, measuring mat, thermometer, stethoscope, measuring tapes, which are used at health facilities every day to assess children.

Neonates born to mothers participating in the WANTAIM trial will be included into the study and followed up at 6 months. Their interest will be protected by way of taking written informed consent. Parents/caregivers will consent on behalf of the child, given the age of the participant involved in the study, at the 72-hour post-natal visit. The parent/caregiver will be informed that their child’s participation is voluntary and that he/she may withdraw the child from the study at any time. Only the researchers will have access to the information obtained from participants. The identity of participants will not be revealed. All participants will be assigned an ID code and this information will always be kept confidential.

# References

1. World Health Organization, Neonatal and Perinatal Mortality, Country, Regional and Global Estimates 2016. [http://apps.who.int/iris/bitstream/handle/10665/43444/9241563206_eng.pdf;jsessi](http://apps.who.int/iris/bitstream/handle/10665/43444/9241563206_eng.pdf%3Bjsessionid%3D2156340D76F40143E459A17865684498?sequence=1) [onid=2156340D76F40143E459A17865684498?sequence=1](http://apps.who.int/iris/bitstream/handle/10665/43444/9241563206_eng.pdf%3Bjsessionid%3D2156340D76F40143E459A17865684498?sequence=1)
2. Wang H, Liddell CA, Coates MM, et al. Global, regional, and national levels of neonatal, infant, and under-5 mortality during 1990-2013: a systematic analysis for the Global Burden of Disease Study 2013. *Lancet* 2014;384:957-79.
3. Kassebaum NJ, Bertozzi-Villa A, Coggeshall MS, et al. Global, regional, and national levels and causes of maternal mortality during 1990-2013: a systematic analysis for the Global Burden of Disease Study 2013. *Lancet* 2014;384:980- 1004.
4. UNICEF for every child alive. The urgent need to end newborn death,2017. https:[//www](http://www.unicef.org/publications/files/Child_Mortality_Report_2017.pdf).u[nicef.org/publications/files/Child_Mortality_Report_2017.pdf](http://www.unicef.org/publications/files/Child_Mortality_Report_2017.pdf)
5. Chauhan SP, Rice MM, Grobman WA, et al. Neonatal Morbidity of Small- and Large-for-Gestational-Age Neonates Born at Term in Uncomplicated Pregnancies. *Obstet Gynecol* 2017:130:511-519.
6. Gladstone M, Oliver C, Van den Broek N. Survival, Morbidity, Growth and Developmental Delay for Babies Born Preterm in Low and Middle Income Countries – Systematic Review of Outcomes Measured. *PLOS ONE* 2015;10:e0120566.
7. Ferusa SA, Harlow SD, Gillespie BW, Welch K, Johnson TR. Birthweight- adjusted Dubowitz methods: reducing misclassification of assessments of gestational age in a Zimbabwean population, [*Cent Afr J Med.*](https://www.ncbi.nlm.nih.gov/pubmed/15214282) 2003;49:47-53.
8. Lee AC, Panchal P, Folger L, et al. Diagnostic Accuracy of Neonatal Assessment for Gestational Age Determination: A Systematic Review. *Pediatrics* 2017;140:e20171423.
9. Van Wyk L, Smith J. Postnatal Foot Length to Determine Gestational Age: A Pilot Study. *J Trop Pediatr* 2016;62:144-151.
10. Thi HN, Khanh DKT, Thu HLT, Thomas EG, Lee KJ, Russell FM. Foot Length, Chest Circumference, and Mid Upper Arm Circumference Are Good Predictors of Low Birth Weight and Prematurity in Ethnic Minority Newborns in Vietnam: A Hospital-Based Observational Study. *PLOS ONE* 2015;10: e0142420. doi:10.1371/journal.pone.0142420.
11. Goto E. Meta-Analysis: Identiﬁcation of Low Birthweight by Other Anthropometric Measurements at Birth in Developing Countries. *J Epidemiol* 2011;21:354-362. doi:10.2188/jea.JE20100182.
12. Elizabeth NL, Christopher OG, Patrick K. Determining an anthropometric surrogate measure for identifying low birth weight babies in Uganda: a hospital- based cross sectional study. *BMC Pediatr* 2013;13:54 <http://www.biomedcentral.com/1471-2431/13/54>
13. Hadush MY, Berhe AH, Medhanyie AA. Foot length, chest and head circumference measurements in detection of Low birth weight neonates in Mekelle, Ethiopia: a hospital based cross sectional study. *BMC Pediatr* 2017; 7:111. doi: 10.1186/s12887-017-08660.
14. KC A, Nelin V, Vitrakoti R, Aryal S, Malqvist M. Validation of the foot length measure as an alternative tool to identify low birth weight and preterm babies in a low-resource setting like Nepal: a cross-sectional study. *BMC Pediatr* 2015; 15:43. doi: 10.1186/s12887-015-0361-4.
15. Pratinidhi AK, Bagade AC, Kakade SV, et al. Action-oriented colour-coded foot length calliper for primary healthcare workers as a proxy for birth weight & gestational period. *Indian J Med Res* 2017;145:347-352. doi: 10.4103/ijmr.IJMR_36_14.
16. Nabiwemba E, Marchant T, Namazzi G, Kadobera D, Waiswa P. Identifying high- risk babies born in the community using foot length measurement at birth in Uganda. *Child Care Health Dev* 2013;39:20-6.
17. Mukherjee S, Roy P, Mitra S, Samanta M, Chatterjee S. Measuring new born foot length to identify small babies in need of extra care: A cross-sectional hospital- based study. *Iran J Pediatr* 2013;23:508-12.
18. Marchant T, Penfold S, Mkumbo E, et al*.* The reliability of a newborn foot length measurement tool used by community volunteers to identify low birth weight or premature babies born at home in Southern Tanzania. *BMC Public Health* 2014;14:859.
19. Marchant T, Jaribu J, Penfold S, Tanner M, Armstrong Schellenberg J. Measuring newborn foot length to identify small babies in need of extracare: a cross sectional hospital-based study with community follow-up in Tanzania. *BMC Public Health* 2010;10:624.
20. Otupiri E, Wobil P, Nguah SB, Hindin MJ. Anthropometric Measurements: Options for Identifying Low Birth Weight Newborns in Kumasi, Ghana. *PLoS ONE* 2014;9:e106712. doi:10.1371/journal.pone.0106712.
21. Chaves MVB, Ximenes CV, Borba SKM, Figueroa JN, Alves JGB. Foot length in newborns small for gestational age. *Trop Doct* 2016;46:156-9. doi: 10.1177/0049475515619511.
22. Linhart C, Carter K, Taylor R, et.al, Mortality Trends in the Pacific Island State. June2014.https://prism.spc.int/images/VitalStatistics/The_Pacific_Report_V35_FI NAL.pdf
23. National Department of Health. National Health Plan 2011-2020: 2014 Sector Performance Annual Review. 2014, Port Moresby, Papua New Guinea.
24. Altman DG. Practical statistics for medical research. 1991, London: Chapman & Hall.

# Appendices

##
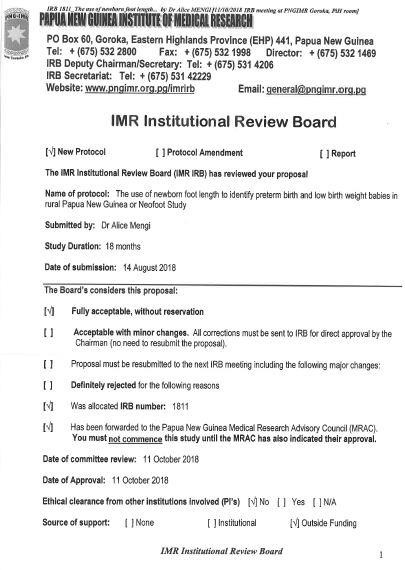
Appendix 1: Ethics Approval from the PNGIMR Institutional Review Board
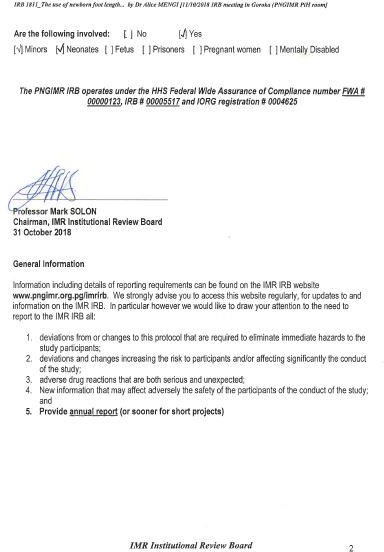


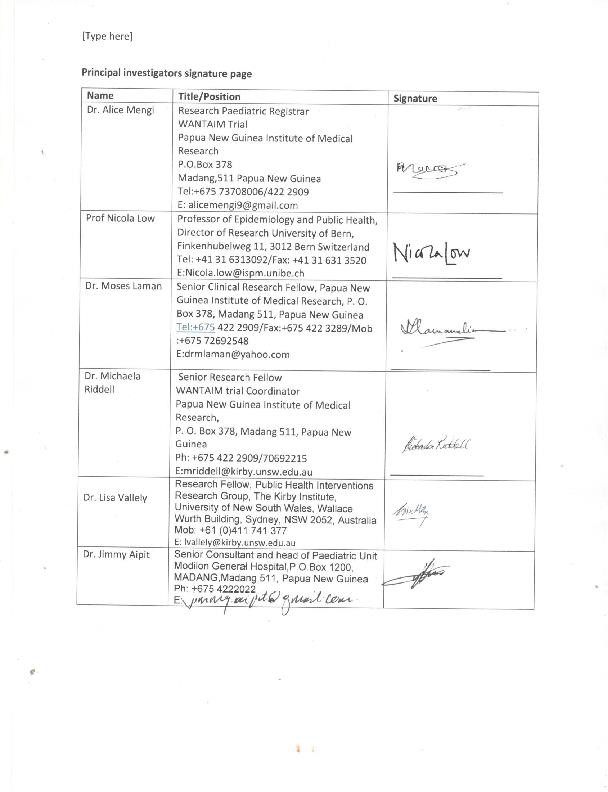


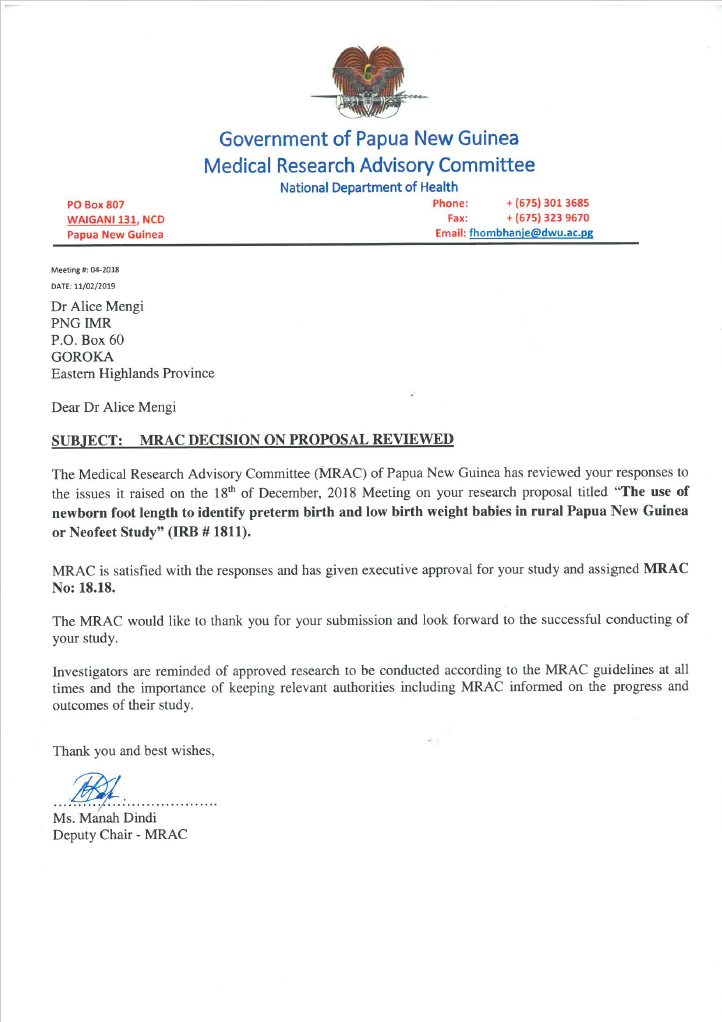


## Appendix 2a. Information sheet (English)


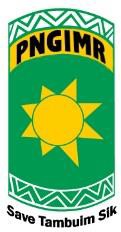
**THE PAPUA NEW GUINEA INSTITUTE OF MEDICAL RESEARCH PARTICIPANT INFORMATION SHEET**

**Full title: The use of newborn foot length to identify preterm birth and low birth weight babies in rural Papua New Guinea (Neofoot study)**

**Please read this information sheet (or ask a friend to read it to you) before you decide to take part. Please ask if there is anything that is not clear.**

**Why is this study being done?**

Most pregnant women in Papua New Guinea, give birth in the villages or a facility without skilled staff and equipment, such as scales, are not always available for staff to assess prematurity and low birth weight. Preterm birth and low birth weight are risk factors for early neonatal death. Thus, this study will potentially provide us with a simple tool that can be used by health workers and lay people to easily identify preterm and low birth weight infants in health facilities and the community. Urgent referral and management of these high-risk infants may reduce the risk of early neonatal death.

**What will happen to your child if you agree to take part in this?**

We are seeing you and your baby now to conduct a post-natal assessment for you and your baby, as part of the main WANTAIM study. Your baby is also going to have follow up visits in 1-2 weeks’ time and 4-6 weeks’ time. In addition to this, we have another post-natal study, which we will go through with you now. We will provide all information and allow you to ask questions. After all your questions are answered and if you agree for your child to join the study, we will ask you to sign the consent form to give your permission. If your cannot read or write, we will ask you to have a witness to be present for the whole of this discussion. After the discussion, if you agree for your child to join the study, we will ask you to put your thumbprint on the consent form and the witness will sign for confirmation.

Then, Dr. Mengi, the WANTAIM doctor and the community reporter will measure the length of the right foot of your baby and the doctor will also measure the circumference of your baby’s right arm and record the results into the case record form.

We would then like to see your baby again at 6 months to measure their foot length, head circumference, mid upper arm circumference, weight and body length. We will ask some questions about immunisation status, about general health and wellbeing of your baby and about your baby’s development. This assessment will be done by the WANTAIM doctor at the health facility when the child returns for 6 month immunisations, or we may come to your home/community to see your baby at that time

**Who can participate in this study?**

All babies of women who enrolled into the main WANTAIM in Madang sites where extended post-natal follow up is taking place are eligible to participate in this study.

**How long will my baby be in the study?**

We will check your baby now and in 6 months, when your baby is due for their 6 month childhood immunisations.

**Do I have to take part?**

No. It is up to you to decide. You can also decline to answer any questions or to change your mind about being part of the study if you want to.

**Can my child stop taking part?**

Yes, you, on behalf of your child, can decide to stop taking part whenever you choose. This would mean that you do not need to explain why you want to stop taking part to anyone, just that you want to stop. This will not affect any care you receive now or in the future.

**What are the risks in participating?**

The risks involved with the study are likely to be minimal or none as the team will use a plastic ruler to measure the foot length. We will use paediatric tape measures to measure your baby’s head circumference. Your baby may exhibit some very mild discomfort during these measurements while holding their feet or head still.

**What are the benefits in taking part in the study?**

Based on this study, the findings can help in policy making and improve health of other children in future.

**What will happen to the information we collect from your child?**

When you agree for your baby to take part in this study, your baby will be assigned a study identification number and it will be used instead of their name so all the information obtained about your baby will kept secret.

Your contact details will be filed separately and only made available to staff members if your baby needs further treatment or referral.

All the information we collect will remain secret and will be stored in locked filing cabinets in our offices. We will **NOT** enter anyone’s name or address into the computer. You will **NOT** be identified in in any report or publications arising from this study.

**What is the cost of participating in the study?**

It will not cost you anything to participate in this study.

**What rights do participants have?**

You have the right to withdraw from the study at any point in time. You have the right not to answer any question at any time. This will not affect any treatment or service provided to you by the antenatal clinic.

**How will I find out about the results of this study?**

After the study has been completed, the results will be analysed as part of Dr Mengi’s Masters qualification through the University of PNG. This can take up to 6-12 months. After this you will be told the results of the overall study. A plain language summary report in *Tok-Pisin* and English will be produced and provided to women who participated in the research, community groups and key stakeholders, including policy makers and health service providers.

The results of the study will also be written up and submitted for review by a medical journal. They may also be presented at national and international meetings and scientific conferences.

You will **NOT** be identified in any conference presentation, report, or medical journal article.

**Who has approved this study?**

This study has been approved by the PNG IMR Institutional Review Board, the Medical Research Advisory Committee in PNG and the University of PNG.

**Who can I speak to if I have questions or problems?**

If you would like any more information about this study please contact:

**Dr,Alice Mengi, Research Paediatric Registrar, PNG IMR, Madang Province, PNG**

Tel: +675 737 08006 / 422 2909 / E: [alicemengi9@gmail.com](mailto:alicemengi9@gmail.com)

**Dr Michaela Riddell, WANTAIM Study Coordinator, PNG IMR, Madang Province, PNG Tel:** Tel: +675 422 2909 / 422 2962 / Fax: +675 422 3289 / E: [mriddell@kirby.unsw.edu.au](mailto:mriddell@kirby.unsw.edu.au) **Dr Moses Laman Senior Clinical Research Fellow, PNG IMR, Madang Province, PNG**,

Tel: +675 422 2909 / 422 2962 / Fax: +675 422 3289 / Mob: +675 7269 2548 / E: [drmlaman@yahoo.com](mailto:drmlaman@yahoo.com)

**Full list of Neonatal Foot Length (Neofoot) study investigators**

| **Investigators** | |
| --- | --- |
| Dr. Alice Mengi | Research Paediatric Registrar, WANTAIM Trial, Papua New Guinea Institute of Medical Research, P.O.Box 378, Madang, 511 Papua New Guinea |
| Prof. Nicola Low | Professor of Epidemiology and Public Health, Director of Research, Institute of Social and Preventive Medicine, University of Bern, Mittelstrasse 43, 3012 Bern, Switzerland |
| Dr. Moses Laman | Senior Clinical Research Fellow, Papua New Guinea Institute of Medical Research,  P.O. Box 378, Madang 511, Papua New Guinea  Adjunct Professor, School of Medicine and Health Sciences, University of Papua New Guinea |
| Dr. Michaela Riddell | Senior Research Fellow, WANTAIM Trial Coordinator, Papua New Guinea Institute of Medical Research, P. O. Box 378, Madang 511, Papua New Guinea |
| Dr. Lisa Vallely | Research Fellow, Public Health Interventions Research Group, The Kirby Institute, University of New South Wales, Wallace Wurth Building, Sydney, NSW 2052, Australia |

You will be given a copy of this form to keep.

## Appendix 2b. Consent form (English)


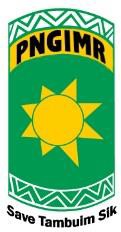
 **THE PAPUA NEW GUINEA INSTITUTE OF MEDICAL RESEARCH**

**Neonatal Foot Length (Neofoot) study consent form**

*You are making a decision whether or not to participate. Your signature indicates that having read the information provided, you have decided to join the study.*

I, (*give name*) have read the participant information sheet, or had the information sheet read to me, and I agree for my child to take part in the study called the **‘Neonatal Foot Length (Neofoot) Study’**

I hereby confirm that:

1. I have been given an opportunity to ask questions about the study and my participation in it.
2. I understand that all the information I provide, examination findings and the results of my tests for my child will be kept strictly confidential and stored in a secure location.
3. I understand that my privacy will be maintained at all times and my child will not be identifiable in any report or publication about this study.
4. I understand that by joining the study I agree to be followed-up at the antenatal clinic or in the community after I have given birth and in 6months.
5. The study procedures have been explained to me and I am willing to take part in this study.
6. I understand that I am free to withdraw from the study at any time, and that if I do so, I can still receive treatment and other services as usual from my local clinic or hospital**Signature or thumbprint of parent/guardian**

*Note to research staff: A thumbprint is to be provided* ***ONLY*** *if a participant cannot read and/or write. Thumbprints* ***MUST*** *be witnessed i.e. an appropriate witness must also sign below for this consent to be considered valid*

| **Signature or thumbprint** | **Date:** |
| --- | --- |
|  |  |

**Signature of witness (if parent/guardian is unable to read or write)**

I, (*please write name*) hereby confirm that the person named above has acquired a full understanding of the research study and has freely consented to participating in the study.

| **Signature** | **Date:** |
| --- | --- |
|  |  |

**Signature of clinic study staff obtaining consent**

I, (*please write name*) hereby confirm that the person named above has acquired a full understanding of the research study and has freely consented to participating in the study.

| **Signature** | **Date:** |
| --- | --- |
|  |  |

## Appendix 3a. Information sheet (Pidgin version)


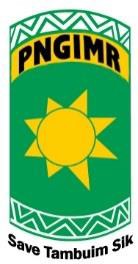
**THE PAPUA NEW GUINEA INSTITUTE OF MEDICAL RESEARCH**

**TOKSAVE PEPA**

**Full title: The use of newborn foot length to identify preterm birth and low birth weight babies in rural Papua New Guinea**

**Plis ridim dispela toksave pepa gut (o askim wanpela lo ridim lo yu) pastaim lo yu kamapim tingting lo stap insait long wok painim aut. Plis askim sapos sampela samting ino klia.**

**Wanem a tingting bilong dispela wok painim aut?**

Planti bel mama insait lo Papua Niu Gini i save karim pikinini lo ples or long ol liklik hausik wei planti taim ino save gat ol masin olsem skel or hevi bilong kisim skel bilong ol beibi na lukim sapos beibi I kamap huriap tumas or skel o hevi bilong beibi i tamblo tumas. Ol beibi I kamap huriap tumas na skel I tamblo tumas,ol i ken painim sik na die.Dispela wok painim aut i ken kamap wantaim gutpela wei we ol wok manmeri bilong hausik na ol lain i save helpim ol mama lo kisim beibi ken usim na luksave sapos beibi skel/hevi i tamblo tumas or kamap huriap tumas. Ol woklain i ken luksave na salim beibi huriap long hausik na beibi i ken kisim halivim na stap laip.

**Wanem samting bai kamap sapos yu tokrait lo beibi bilong yu long stap insait long wok painim aut?**

Beibi bilong yu i stap long wok painim aut bilong ol beibi, na ba mipela askim yu long kisim beibi kam gen lo sekim skel lo 1-2wik na 4-6wiks taim. Beibi bilong yu tu I ken stap long niupela wok painim aut.

Mipela bai stori moa wantaim yu nau.Bai mipela i stori moa lo wonem samting mipela ba wokim na seim taim tu yu ken askim sapos yu no kilea gut lo wonem samting mipela stori. Behain lo yu kisim olgeta stori na mipela bekim olgeta askim bilong yu na sapos yu amamas lo beibi bilong yu joinim wok painimaut, orait bai yu signim tokorait pepa long givim tok orait bilong yu. Sapos yu no save long rit na rait bai mipela askim lo kisim wanpela witness lo stap taim mipela stori long yu long dispela wok painim aut. Behain long olgeta stori na yu amamas lo beibi bilong yu long kam insait long wok painim, ba yu putim pinga mak na witness bilong yu bai sainim tok orait pepa.

Bihain lo yu givim tokorait, docta bai i makim longpela bilong rait lek bilong beibi na makim raunim rait han bilong beibi aninit lo solda. Na ol community ripota/voluntia tu bai makim longpela bilong rait lek bilong beibi bilong nau. Na long six mun taim bai docta i sekim beibi gen taim yu kisim beibi I kam long kisim 6mun bebi sut o kam sekim yu long haus.Bai docta i makim longpela bilong rait lek bilong beibi, makim raunim rait han aninit lo solda, kisim kilo bilong beibi,longpela bilong beibi na makim raunim het bilong beibi. Ba mi kisim tu galas bilong beibei, sekim sopas hat bilong beibi i pump gut na kisim kautim long wanpela minit, sekim sapos beibi I pulim win gut and kautim long wanpela minute na sekim bebi sapos em grow gut. Mipela bai askim sampela moa askim lo sait bilong beibi sut, sapos beibi I stap gut na sekim sapos beibi I stap orait tasol.

**Husait i ken stap insait long wok painim aut?**

Olgeta beibi bilong ol mama bilong i stap insait long WANTAIM study insait lo Madang.

**Beibi bilong mi bai stap insait long wok painim aut inap long amas wik,mun or yia?**

Sapos beibi bilong yu stap insait long wok painim aut bai mipla sekim em nau na long 6mun taim na bai pinis long wok painim aut.

**Sapos mi les long beibi bilong long stap insait long wok painim aut?**

Em orait.Emi laik bilong yu. Yu ken tok nogat long bekim ol askim o long senisim tingting bilong yu long stap insait long wok painim aut sapos yu laik.

Dispela yu no nid long toksave long wanpela wanem as yu laik stop long wok painim aut, i olsem yu yet yu laik stop. Dispela bai no inap bagarapim wanpela halivim yu kisim nau o long bihain taim.

**Wanem sampela hevi beibi bilong mi ken bungim sapos em i stap insait long wok painim aut?**

I nogat birua i stap insait long dispela wok painim aut. Mipela i usim rula,tape mesa,skel, temomita na stetaskop. Em ol dispela samting wei ol wokmameri lo hausik i save usim olgeta dei long klinik lo sekim sapos beibi i stap orait.

**Wanem sampela gutpela samting long stap insait long wok painim aut?**

Sapos beibi bilong yu I stap insait long wok painim nau, em i ken halivim lo savim ol narapela beibi i kamap behain taim sapos dispela wok painim aut I soim wanpela gutpela wei long luksave na halivim beibi i kamap huriap tumas and skel I tambilo tumas.

Seim taim tu displa wokpainim aut em kamap osem skul wok bilong Dr. Mengi husait i wok wantaim PNGIMR na ba skul long UPNG.

**Olgeta stori na tes bilong mi bai stap hait?**

Yes. Wokman bilong clinic tasol bai save long fone namaba o wonem hap yu stap.Wokman husait i lukautim dispela wok painim aut,bai sekim tokorait pepa na bai lukim nem bilong yu. Olgeta narapela stori bai istap hait na i no inap long usim nem bilong yu insait long wok painim aut. Bai yu usim wanpela namba insait long wok painim aut wei ba I haitim olgeta stori na tes bilong yu.

Olgeta stori na tes bilong yu bai I stap hait insait long wanpela kebinet long office bilong mipela. Dispela ol stori na tes bai mipela putim go insait lo komputa wantaim namba tasol. Mipela bai no inap putim nem bilong yu o bai nem biliong no inap kamap ples klia taim mipela I raitim report o stori long ol save manmeri insait long ol bikpla bung long dispela wok painim aut.

**Hamas long stap insait long wok painim aut?**

Ino gat kos long beibi bilong yu long stap insait long wok painim aut.Olgeta sek em free.

**Wanem raits bilong ol beibi husait stap insait?**

Yu gat rait sapos yu laikim beibi bilong yu long lusim wok painim aut long wanem taim yu laik.Yu gat rait long noken bekim askim long eni taim.Dispela bai no inap banisim yu long kisim halvim long bel-mama klinik.

**Hau bai mi save long risals bilong wok painim aut?**.

Bihain long dispela wok painim aut i pinis long 6-12 mun igo pinis. Ba mipela raitim wanpela liklik report long dispela wok painim aut long tok pisin an inglis. Dispela ripot bai mipela I go insait long ol komunity wei ol papamama na o lukaut manmeri bilong ol bebi i stap long em.Seim taim tu dispela ripot bai igo long lain I save go pas wokim ol sait bilong helt insait long provins na kauntry.

Ol bai ritim olgeta samting ol I painim insait long dispela wok painim aut go insait long bikpla ripot bilong medical jenel na tok aut long bikpla bung insait long kauntry na outsait long ol narapela kauntry tu.

Yu bai **no inap** kamap ples klia insait long bikpla bung na ripot.

**Husait i givim tok orait long dispela stadi?**

PNG IMR Institutional Review Board na Medical Research Advisory Committee insait long PNG i givim tok orait long dispela stadi.

**Bai mi toktok long husait sapos mi gat askim o hevi?**

Sapos yu laikim moa toksave long dispela stadi plis kontek:

**Dr,Alice Mengi,Research Paediatric Registrar Papua New Guinea Institute of Medical Research Tel:** +675 737 08006 / 422 2909 / E: [alicemengi9@gmail.com](mailto:alicemengi9@gmail.com)

**Dr Michaela Riddell, Study Coordinator, PNG IMR, Madang Province PNG**

**Tel:** Tel: +675 422 2909 / 422 2962 / Fax: +675 422 3289 / E: [mriddell@kirby.unsw.edu.au](mailto:mriddell@kirby.unsw.edu.au)

**Dr Moses Laman Senior Clinical Research Fellow, PNG IMR, Madang Province PNG**,

**Tel:** +675 422 2909 / 422 2962 / Fax: +675 422 3289 / Mob: +675 7269 2548 / E: [drmlaman@yahoo.com](mailto:drmlaman@yahoo.com)

**Ol manmeri husait I go pas long dispela wok painim aut**

| **Investigators** | |
| --- | --- |
| Dr. Alice Mengi | Research Paediatric Registrar WANTAIM Trial  Papua New Guinea Institute of Medical Research P.O.Box 378  Madang,511 Papua New Guinea |
| Prof Nicola Low | Professor of Epidemiology and Public Health, Director of Research, Institute of Social and Preventive Medicine, University of Bern, Mittelstrasse 43, 3012 Bern Switzerland |
| Dr. Moses Laman | Senior Clinical Research Fellow, Papua New Guinea Institute of Medical Research, P. O. Box 378, Madang 511, Papua New Guinea  Adjunct Professor, School of Medicine and Health Sciences, University of Papua New Guinea |
| Dr. Michaela Riddell | Senior Research Fellow WANTAIM trial Coordinator  Papua New Guinea Institute of Medical Research,  P. O. Box 378, Madang 511, Papua New Guinea |
| Dr. Lisa Vallely | Research Fellow, Public Health Interventions Research Group, The Kirby Institute, University of New South Wales, Wallace Wurth Building, Sydney, NSW 2052, Australia |

Bai yu kisim wanpela kopi bilong dispela pepa.

## Appendix 3b. Consent form (Pidgin)


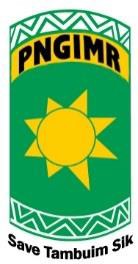
**THE PAPUA NEW GUINEA INSTITUTE OF MEDICAL RESEARCH**

**Neonatal foot length (Neofoot) study: Tok orait pepa**

*Yu givim tok orait bilong yu long stap o, noken stap insait long dispela wok painim aut..Han mak bilong yu i soim olsem bihain long yu ridim dispela toksave, yu laik stap insait long dispela wok painim aut.*

Mi, (*raitim nem long hia*) ridim pinis dispela wokbung wantaim toksave pepa, o wanpela i ridim toksave pepa long me, na mi wanbel long stap insait long wok painim aut ol i kolim **‘Neonatal foot length (Neofoot) study’**

Mi nau tok stret olsem:

1. Mi bin gat taim long askim sampela askim long dispela wok painim aut
2. Mi save olsem olgeta stori mi givim na ol tes risal bilong mi bai stap hait long gutpela lukaut.
3. Mi save olsem olgeta stori bilong mi bai stap hait na mi bai stap gut long olgeta taim na me no inap kamap ples klia insait long ol ripot bilong dispela wok painim aut.
4. Mi save olsem bai yupela i makim longpela bilong rait leg and raunpela bilong right han uninit lo solda.
5. Mi save osem bai mi kisim beibi kam bek lo 6mun taim gen. Ba ol i makim longpela bilong rait leg and raunpela bilong right han uninit lo solda,kisim kilo bilong beibi, makim longpela bilong beibi,makim raunpela bilong het bilong beibi. Sem taim bai ol I sekim sapos beibi i stap orait tasol na kisim olgeta beibi sut.
6. Mi save olsem beibi bilong mi iken lusim stadi long wanem taim mi laik, na sapos mi mekim olsem, mi bai kism ol narapela halivim yet long klinik bilong o hausik klostu long mi.

**Hanmak bilong papa mama or husait i lukautim beibi**

*Sapos mama na papa or husait I lukautim beibi I no save long rit na rait orait em ba putim pinga mak tamblo na wintness ba i ken putim hanmak(sain) bilon em tu.*

| **Han mak o pinga mak** | **Deit:** |
| --- | --- |
|  |  |

**Nem na hanmak bilong witnes (sapos papa mama/wasmanmeri bilong ino save lo rit na rait)**

Mi, (*plis raitm nem*) tok stret olsem dispela man/meri wantaim nem antap i kisim olgeta save long olgeta stori bilong wok painim aut na itok orait long beibi bilong em long stap insait long wok painim aut.

| **Han mak:** | **Deit:** |
| --- | --- |
|  |  |

**Nem na hanmak bilong wok manmeri**

Mi, (*plis raitim nem*) ) tok stret olsem dispela man/meri wantaim nem antap i kisim olgeta save long olgeta stori bilong wok painim aut na itok orait long beibi bilong em long stap insait long wok painim aut.

| **Hanmak:** | **Deit:** |
| --- | --- |
|  |  |
