# Supplementary material for: The use of newborn foot length to identify low birth weight and preterm babies in Papua New Guinea: A diagnostic accuracy study
Source: PLOS Glob Public Health. 2023 Jun 21;3(6):e0001924. doi: 10.1371/journal.pgph.0001924 (PMC10284404; doi:10.1371/journal.pgph.0001924)
Supplement: S1 Data — (DOCX) [file pgph.0001924.s007.docx]

Neofoot study. The use of newborn foot length to identify low birth weight and preterm babies in Papua New Guinea: A diagnostic accuracy study

# Data

The data that were used to produce Table 1, Table 2, Figures 1-5, S1 Fig, S2 Fig are available at the Neofoot project page on the Open Science Framework <https://osf.io/zjtcn/>. The data are published under a CC-By Attribution 4.0 International license.

The data used to produce Table 3 are available in S1 Table and S2 Table.
