# Supplementary material for: The use of newborn foot length to identify low birth weight and preterm babies in Papua New Guinea: A diagnostic accuracy study
Source: PLOS Glob Public Health. 2023 Jun 21;3(6):e0001924. doi: 10.1371/journal.pgph.0001924 (PMC10284404; doi:10.1371/journal.pgph.0001924)
Supplement: Attachment — Submitted filename: Rebuttal_letter_v1_230421.pdf [file pgph.0001924.s008.pdf]

UNIVERSITÄT
BERN

Universität Bern, Hochschulstrasse 6, 3012 Bern

Faculty of Medicine
Institute of Social and
Preventive Medicine

Mittelstrasse 43
3012 Bern
+41 31 684 35 11
https://www.ispm.unibe.ch

Nicola Low, DTM&H MSc MD FFPH
Professor of Epidemiology and Public Health
+41 31 684 30 92
+41 79 419 37 81
nicola.low@unibe.ch

Prof. Catherine Kyobutungi and Prof. Madhukar Pai,
Editors-in-Chief,
PLOS Global Public Health

Bern, 16 April, 2023

Re: PGPH-D-23-00319R1, response to reviewers

Dear Prof. Kyobutungi and Prof. Pai,

Please find below our point-by-point response to all comments, which we have numbered. Where we have made changes in the text, we give the new wording and the location in the revised version (section and line numbers). We have also uploaded a version with tracked changes, as requested. We hope that these changes address all concerns and that our revised manuscript is of the standard required for publication in PLOS Global Public Health.

Editor's comments

1. In addition to the comments by the two reviewers, I think it will be helpful to explain the context better. When babies are born in health facilities, presumably, actual weight can be obtained and there is no real need to resort to foot length as a proxy. It is when babies are born at home or in remote areas, then surrogates like foot length might be helpful? Surely, we want more women to be born in facilities and we want babies to be actually weighed. So, it is important to make sure that message is clearly conveyed.

Authors' response: We agree with the editor that the ideal situation is for all pregnant women to have the opportunity to give birth in a place where their baby can be weighed. Pregnant women also need to have been assessed during pregnancy to give an accurate date of gestational age at birth; both are needed to identify low birth weight and prematurity. We have made the following changes to give the contextual information and make the message clear:

Abstract, line 37-38, "When birth at a health facility is not possible, foot length measurement can identify LBW and PTB in newborns…"

Introduction, paragraph 1, lines 48-49, "Many babies are, however, not weighed at birth [5] or their mothers have not had an adequate assessment during pregnancy to allow gestational age at birth to be calculated [2]."

Introduction, paragraph 3, lines 65-69, “Although supervised delivery at a health facility is encouraged, uptake of antenatal care in PNG is poor and high-risk babies cannot be easily identified and referred [21,23]. From 2016-2020, only about half of pregnant women attended at least one antenatal visit and just over a third gave birth in a health facility. In Madang Province, about a third of pregnant women had at least one antenatal visit and about a quarter gave birth in a health facility [21].”
Discussion, Implications for practice, lines 334-335, “The goal of universal health coverage to ensure that all sick and small newborns survive and thrive will take time to achieve; in the meantime, there is a need for innovative ways to simplify assessment of gestational age at birth [2].”
Discussion, Implications for practice, lines 339-345, “Most of the women who did not give birth in a health facility lived in hamlets, which are an hour or more from the main road by foot and from which access to the health centre is difficult, especially at night. In settings such as these, validation of newborn foot length as a surrogate for LBW and PTB is important.”

2. In the above context, most babies in this study were born in health facilities and would have had easy access to actual weighing scales. Would it not be necessary to validate these findings in babies born at homes? Isn't that the intended use population? This limitation needs more discussion.
Authors’ response: We agree and we have now added that our study setting was not typical and incorporated the reviewer’s recommendation:

Discussion, Comparison with other studies, lines 297-8, “Both studies were nested in randomised controlled trials,”

Discussion, Comparison with other studies, lines 310-313, “Given the study setting, the proportion of supervised deliveries in our study population was higher than the average for Madang Province [21]. Our study could be repeated among women who do not give birth at a health facility to assess the performance of foot length measurement by a community health worker.”

Reviewer #1

3. I think the authors did a great job explaining every section of the paper. The aim/objective, methods, results section was very well presented; easy to understand. However, the authors could have written more for the discussion and explained their findings more than they have.

Authors’ reply: We thank the reviewer for their positive comments and their observation. We hope that the additional context that we have given to the Editor and to Reviewer #2 give the reviewer the information and explanations that they were seeking.

Reviewer #2

4. The authors have conducted a very important, detailed and nuanced study. The analysis is thorough, and the paper is well written. I would advise a minor revision to adjust the discussion section subheadings to: 1) Summary of main findings 2) interpretation of the findings 3) comparison with other studies 4) strengths and limitations 5) implications for research and practice

Authors’ reply: We thank the reviewer for their suggestion. We followed the structure that Michael Docherty and Richard Smith published (The case for structuring the discussion of scientific papers. BMJ 1999;318:1224-25). They propose that, following the summary of the main findings, “Next should come a

comprehensive examination of the strengths and weaknesses of the study, with equal emphasis given to both. Indeed, editors and readers are likely to be most interested in the weaknesses of the study: all medical studies have them." We hope the reviewer accepts our reasons for structuring the discussion as we wrote it.

On behalf of all the co-authors, I look forward to hearing from you.

Kind regards,

Dr. Nicola Low, DTM&H MSc MD FFPH
Professor of Epidemiology and Public Health
